# Supplementary figures and images for: A machine learning classifier trained on cancer transcriptomes detects NF1 inactivation signal in glioblastoma
Source: BMC Genomics. 2017 Feb 6;18:127. doi: 10.1186/s12864-017-3519-7 (PMC5292791; doi:10.1186/s12864-017-3519-7)

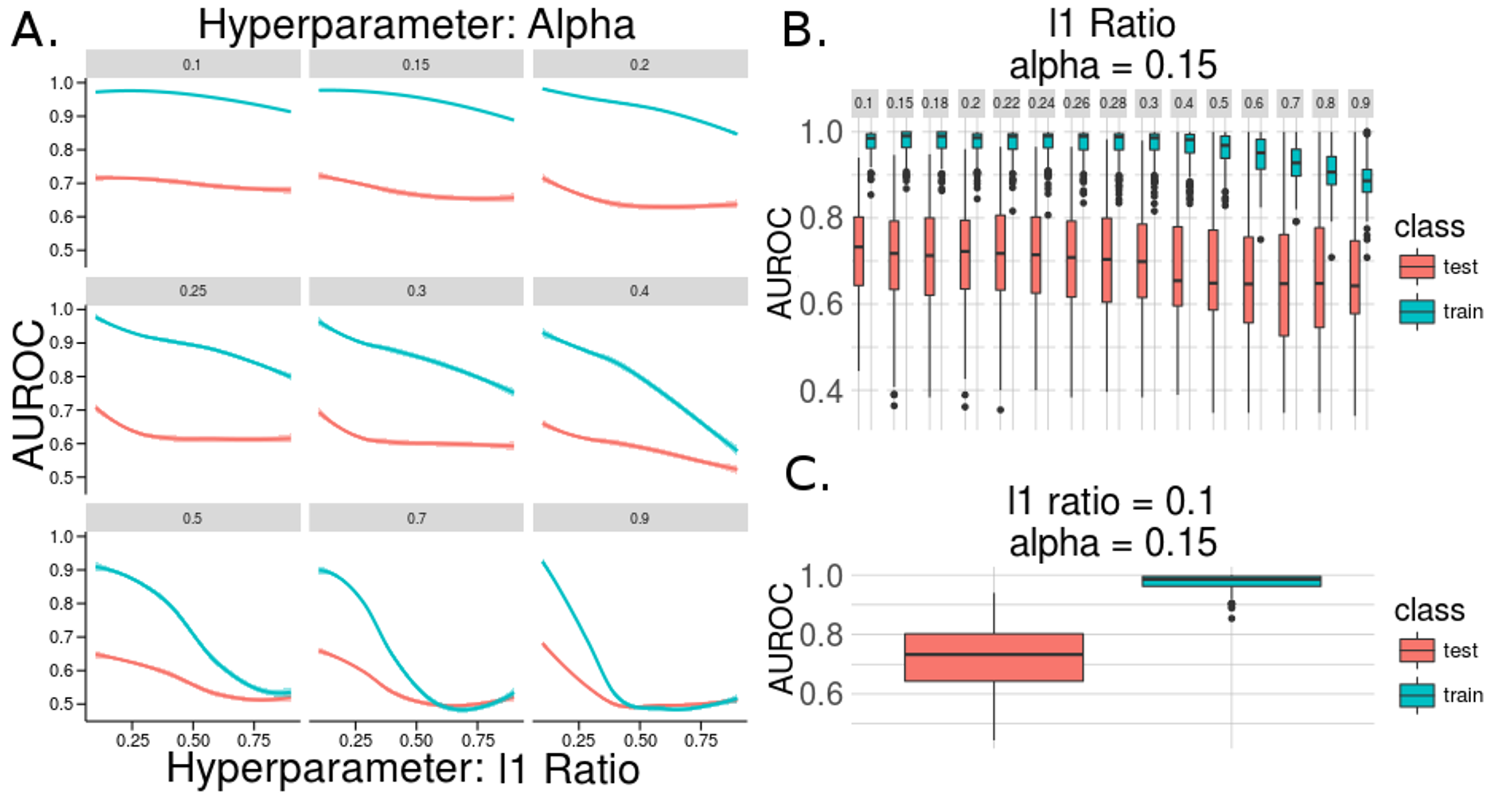

Supplement: Additional file 2: Figure S1. — Non-transformed RNAseq results of The Cancer Genome Atlas Glioblastoma parameter sweep for stochastic gradient descent logistic classifiers with elastic net penalty. (A) Training and testing area under the receiver operating characteristic curve (AUROC) is given for each parameter tested. All accuracies are presented following 5-fold cross validation after 50 random initializations. (B) The l1 mixing parameter with the optimal alpha and (C) the classifier performance across all random starts for the best hyperparameters. (PNG 711 kb) [file 12864_2017_3519_MOESM2_ESM.png]

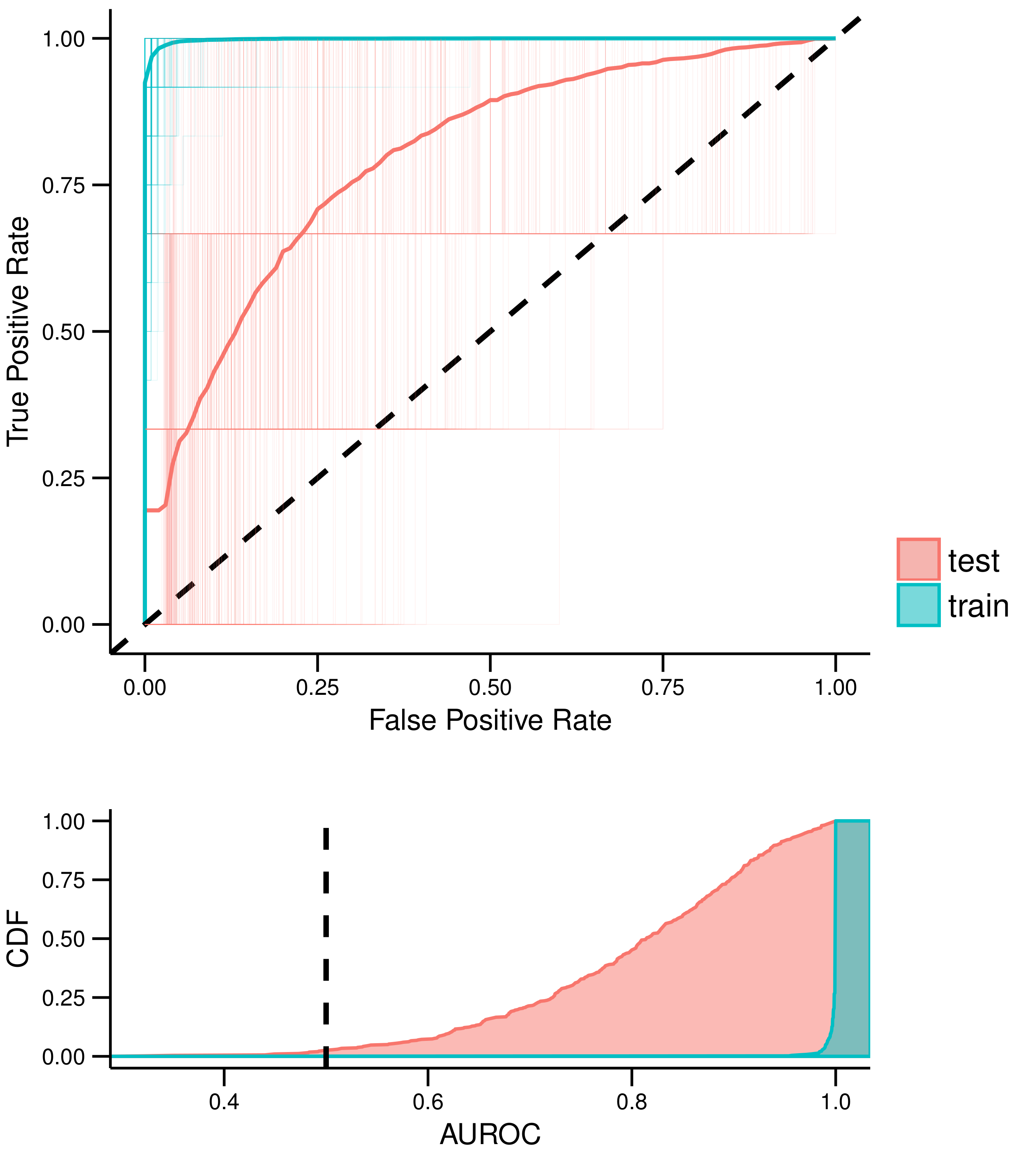

Supplement: Additional file 3: Figure S2. — Logistic regression classifier with elastic net penalty training and testing errors over 100 iterations for non-transformed The Cancer Genome Atlas Glioblastoma RNAseq data. (A) Receiver operating characteristic (ROC) curve and shows the average training and testing performance of 5-fold cross validation over 100 random initializations as well as each individual classifier in the ensemble model. (B) The cumulative density of area under the ROC curve (AUROC) for all training and testing partitions. (PNG 240 kb) [file 12864_2017_3519_MOESM3_ESM.png]

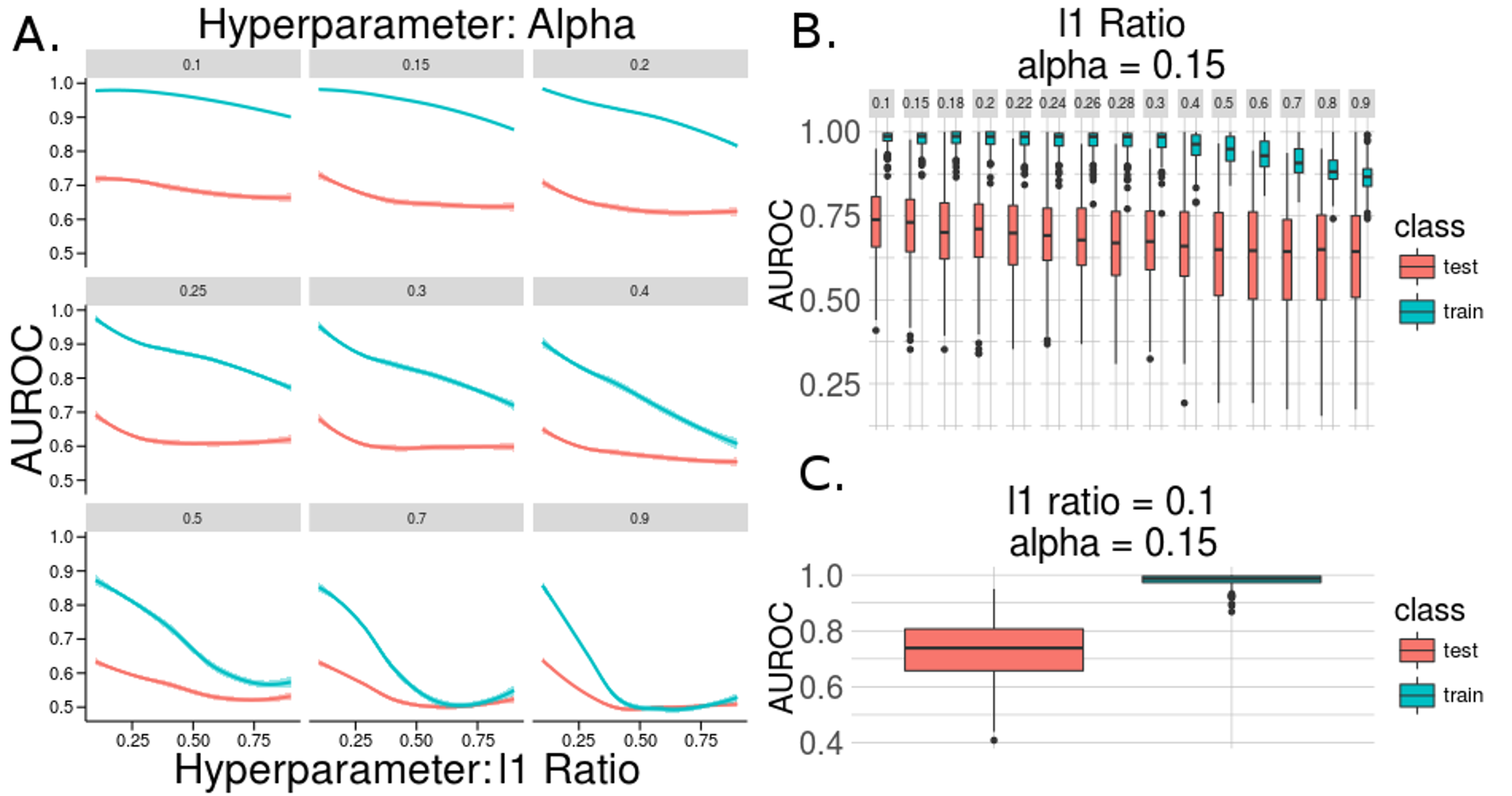

Supplement: Additional file 4: Figure S3. — Training Distribution Matching (TDM) transformation of RNAseq results of The Cancer Genome Atlas Glioblastoma parameter sweep for stochastic gradient descent logistic classifier with elastic net penalty. (A) Training and testing area under the receiver operating characteristic curve (AUROC) is given for each parameter tested. All accuracies are presented following 5-fold cross validation after 100 random initializations. (B) The l1 mixing parameter with the optimal alpha and (C) the classifier performance across all random starts for the best hyperparameters. (PNG 724 kb) [file 12864_2017_3519_MOESM4_ESM.png]

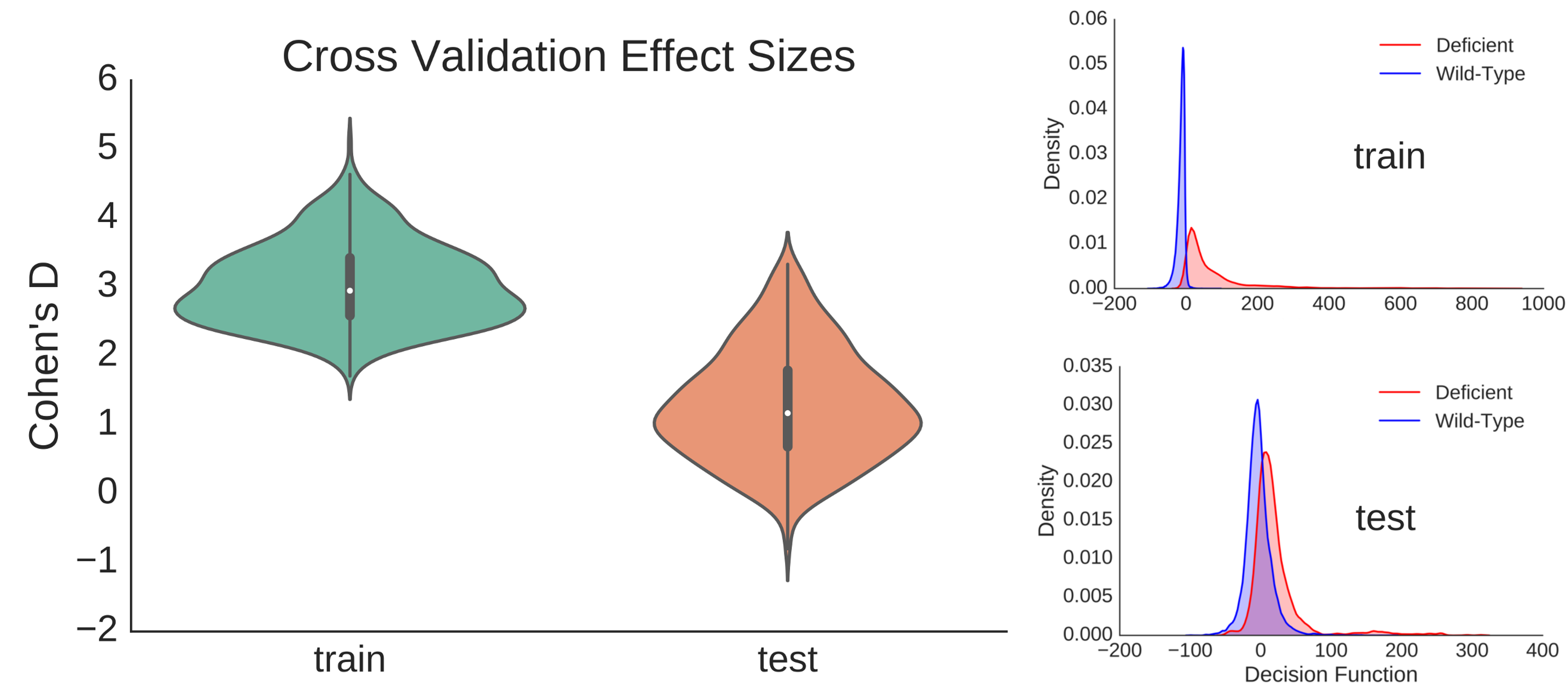

Supplement: Additional file 5: Figure S4. — Cohen’s D effect size estimates across five fold cross validation parameters for all 100 iterations of the TDM transformed ensemble classifier. The effect size for the test set is consistently lower than the training set (left). Additionally, the training and testing decision functions for gold standard NF1 deficient vs. NF1 wildtype samples shows a difference in mean estimates (right). The decision function represents the raw score of all samples as applied to the respective classifiers through each of the 100 iterations of five fold cross validation on the TCGA training set. (PNG 580 kb) [file 12864_2017_3519_MOESM5_ESM.png]
